# Supplementary figures and images for: Allele-specific RNA imaging shows that allelic imbalances can arise in tissues through transcriptional bursting
Source: PLoS Genet. 2019 Jan 9;15(1):e1007874. doi: 10.1371/journal.pgen.1007874 (PMC6342324; doi:10.1371/journal.pgen.1007874)

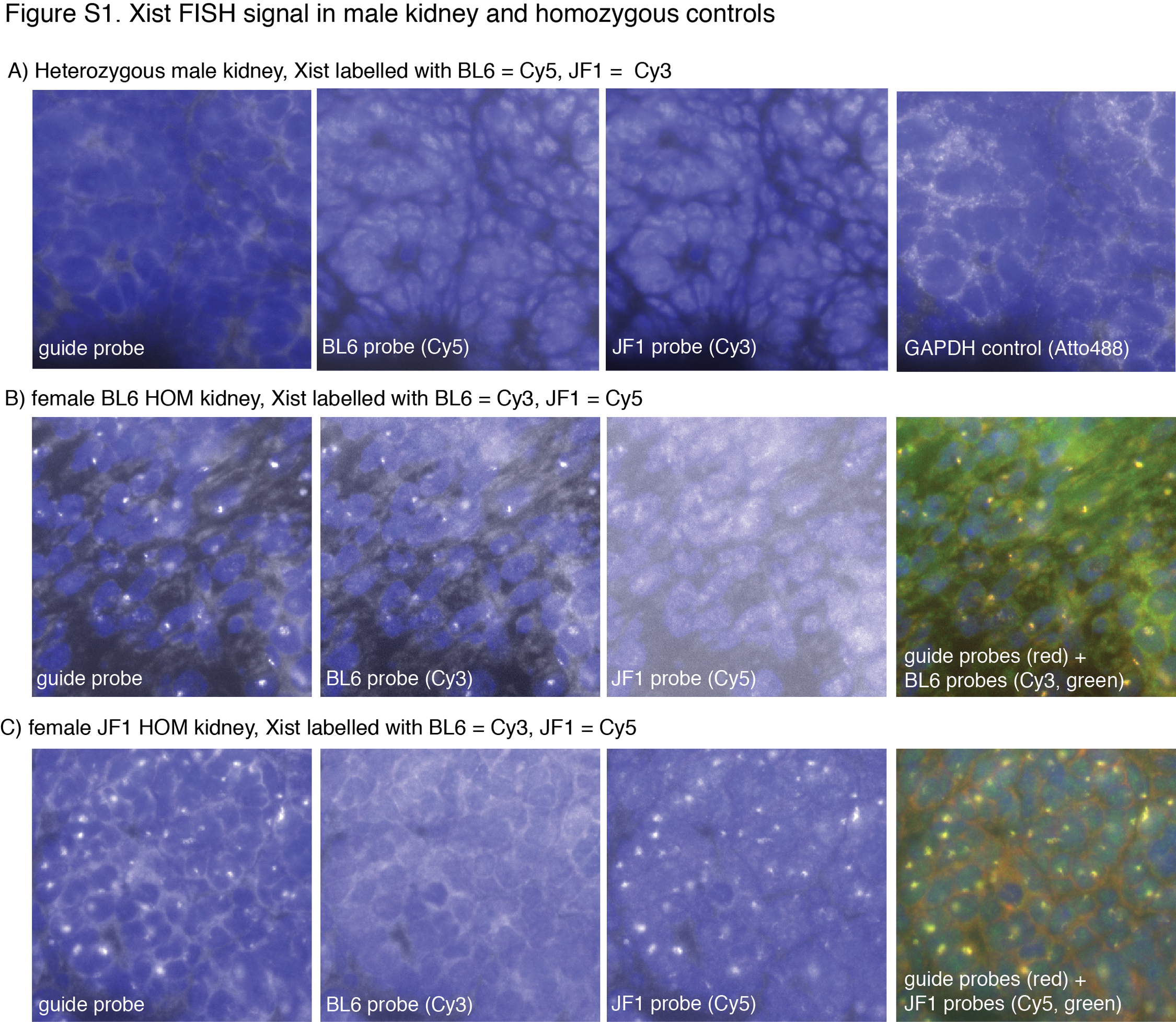

Supplement: S1 Fig — A. Male heterozygous (BL6 x JF1) tissue was stained with probes for Xist (guide probe labelled with Cal fluor 610 (far left), BL6-specific probes labelled with Cy5 (middle left), JF1-specific probes with Cy3 (middle right)), none of which showed signal above background. Atto 488-labelled Gapdh probes were also included to verify absence of RNA degradation (far right). B, C. Female BL6 homozygous (B) and JF1 homozygous (C) tissues were stained with probes for Xist (guide probe labelled with Cal fluor 610 (far left), BL6-specific probes labelled with Cy3 (middle left), JF1-specific probes with Cy5 (middle right)). Right-most image shows colocalization between guide foci and allele-specific foci as indicated. (TIF) [file pgen.1007874.s001.tif]

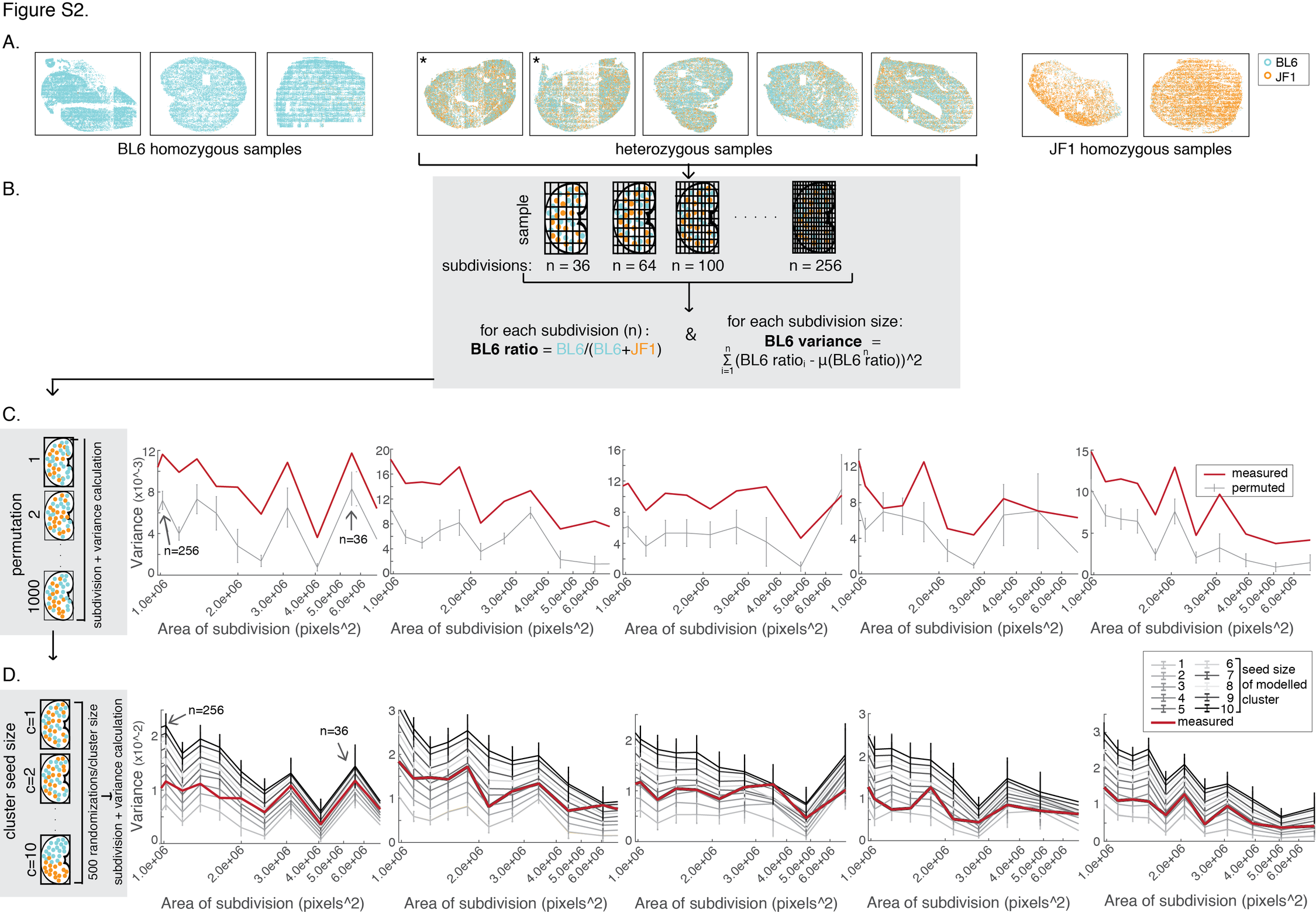

Supplement: S2 Fig — A. Allelic assignments across different BL6 and JF1 homozygous (far left and far right) as well as heterozygous (middle) kidney sections. Two of the heterozygous kidney sections are technical replicates (different kidney sections from the same animal), which is indicated by an asterisk (“*”). BL6 allelic assignment is depicted in turquoise, JF1 allelic assignment is depicted in orange. B. For all heterozygous samples we calculated spatial heterogeneity using a variance metric, the method of which is schematized: sections were subdivided into a grid, using increasingly smaller squares (from 8x8 to 16x16) and for each subdivision we calculated the ratio of BL6 Xist foci. For each grid we then also calculated the variance of the BL6 ratio across all squares of that grid. C. The measured variance (red line) was compared to the variances obtained for samples where we randomly permuted allelic assignments 1000 times (black line, error bars representing standard deviation of the modeled results). The graphs show the variance for subdivisions of different sizes, with both the area of the subdivisions and the size of the grid indicated. D. Measured variance (red line) was also compared to the variances of samples where we randomly placed different sized clusters (seeds) of allelically identical Xist foci in the tissue (lines in different shades of grey, error bars representing standard deviation of the modeled results). For each seed size we generated 500 randomizations, keeping the allelic ratio constant. For all heterozygous data shown in A, C and D the order of the samples is kept identical. (TIF) [file pgen.1007874.s002.tif]

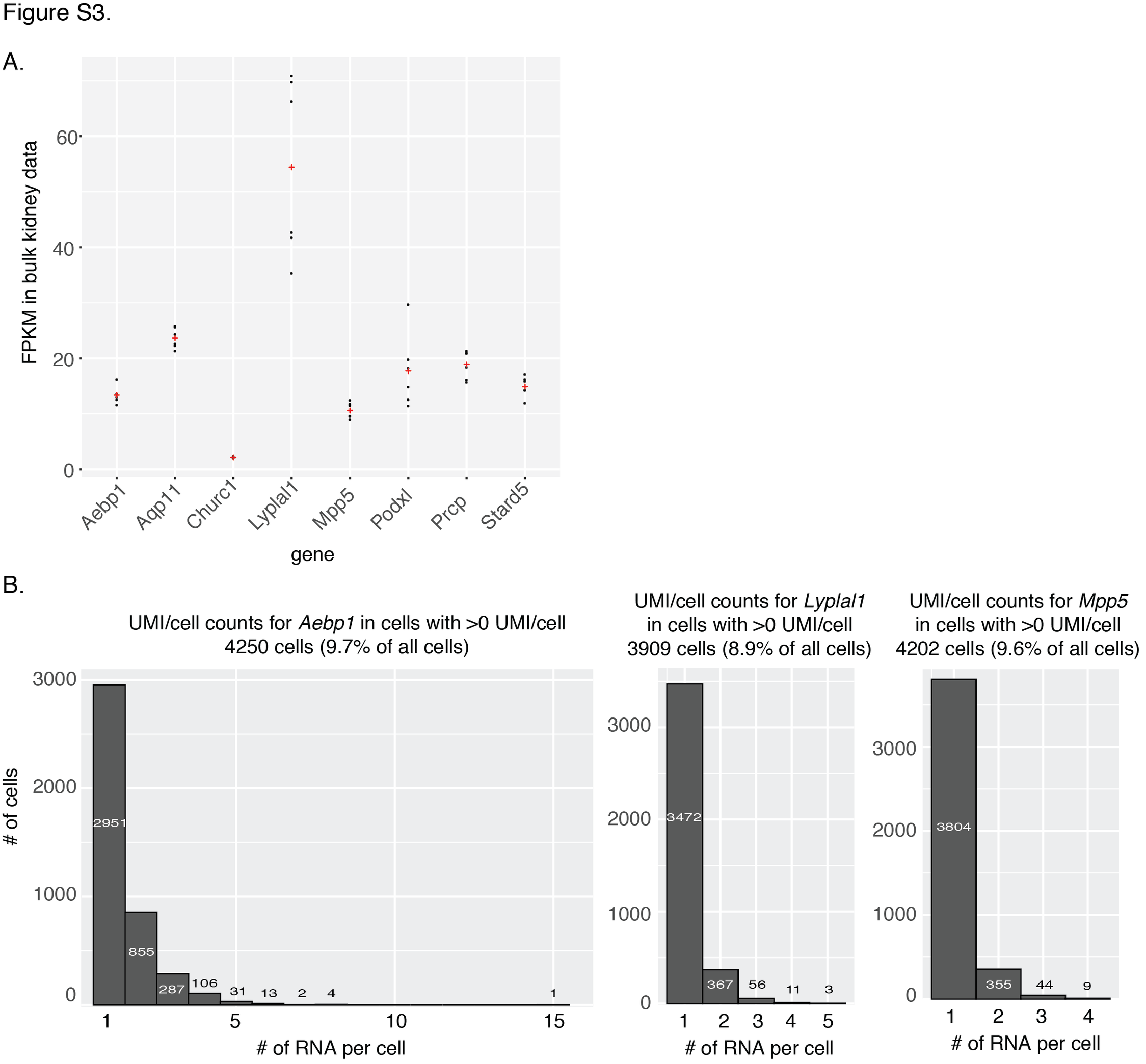

Supplement: S3 Fig — A. FPKM values of six control samples from Beckerman et al [79] are shown for the genes used in this study. Red crosses shown the mean of these values. B. UMI counts per cell for Aebp1, Lyplal1 and Mpp5 for cells with non-zero UMIs, based on data from Park et al [62]. (TIF) [file pgen.1007874.s003.tif]

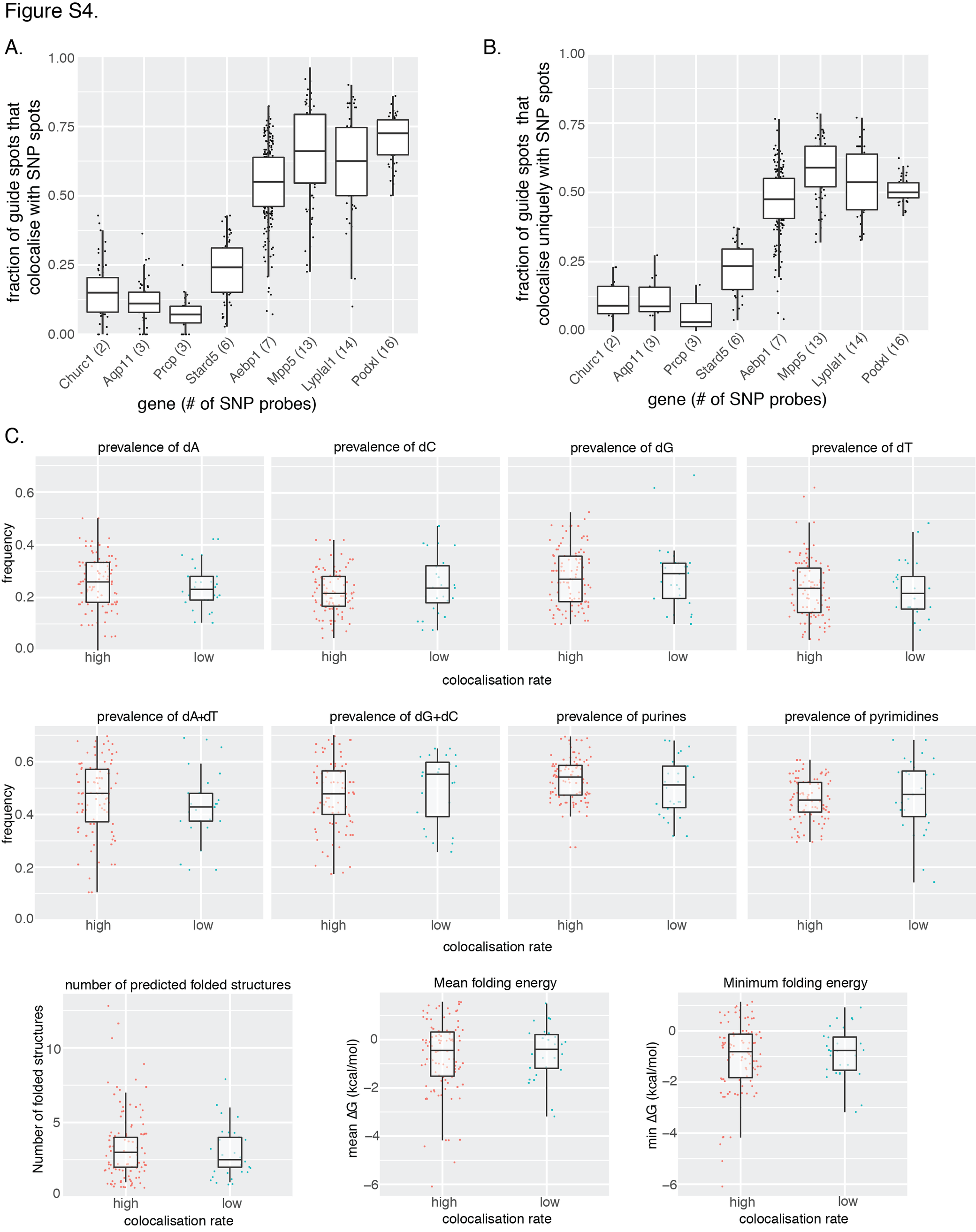

Supplement: S4 Fig — A, B. Overall (A) and allele-specific (B) colocalization rates for different autosomal genes. Overall colocalization rates consider all guide spots that colocalize with either BL6 and/or JF1/C7 allele-specific signal, while allele-specific colocalization counts only those guide spots that colocalize uniquely with either BL6 or JF1/C7 probes. Each spot represent the colocalization rate in one area tested (typically 10–50 cells). All genes were detected with guide probes labelled with Cal fluor 610, and the following allele-specific probes: Aebp1, Mpp5 and Podxl BL6-specific probes labelled with Cy3, JF1-specific probes labelled with Cy5; Churc1 and Lyplal1 BL6-specific probes labelled with Cy5, JF1-specific probes labelled with Cy3; Aqp11 and Stard5 BL6-specific probes labelled with Cy3, probes for the C7 allele labelled with Cy5; Prcp BL6-specific probes labelled with Cy5, probes for the C7 allele labelled with Cy3. Genes are listed in increasing order of number of SNV probes utilized, which is indicated for each gene. C. Probe properties for probe sets with high (>50%) and low (<50%) mean overall colocalization rate. We compared prevalence of individual nucleotides (dA, dC, dG, dT—top row), nucleotides forming three hydrogen bonds (dC+dG) or two hydrogen bonds (dA+dT), purines (dA+dG) and pyrimidines (dC+dT) (middle row), as well as the number of folded structures predicted for each probe, mean and minimum folding energy for each probe (bottom row). For all plots, each spot represents the value obtained for a single probe. (TIF) [file pgen.1007874.s004.tif]

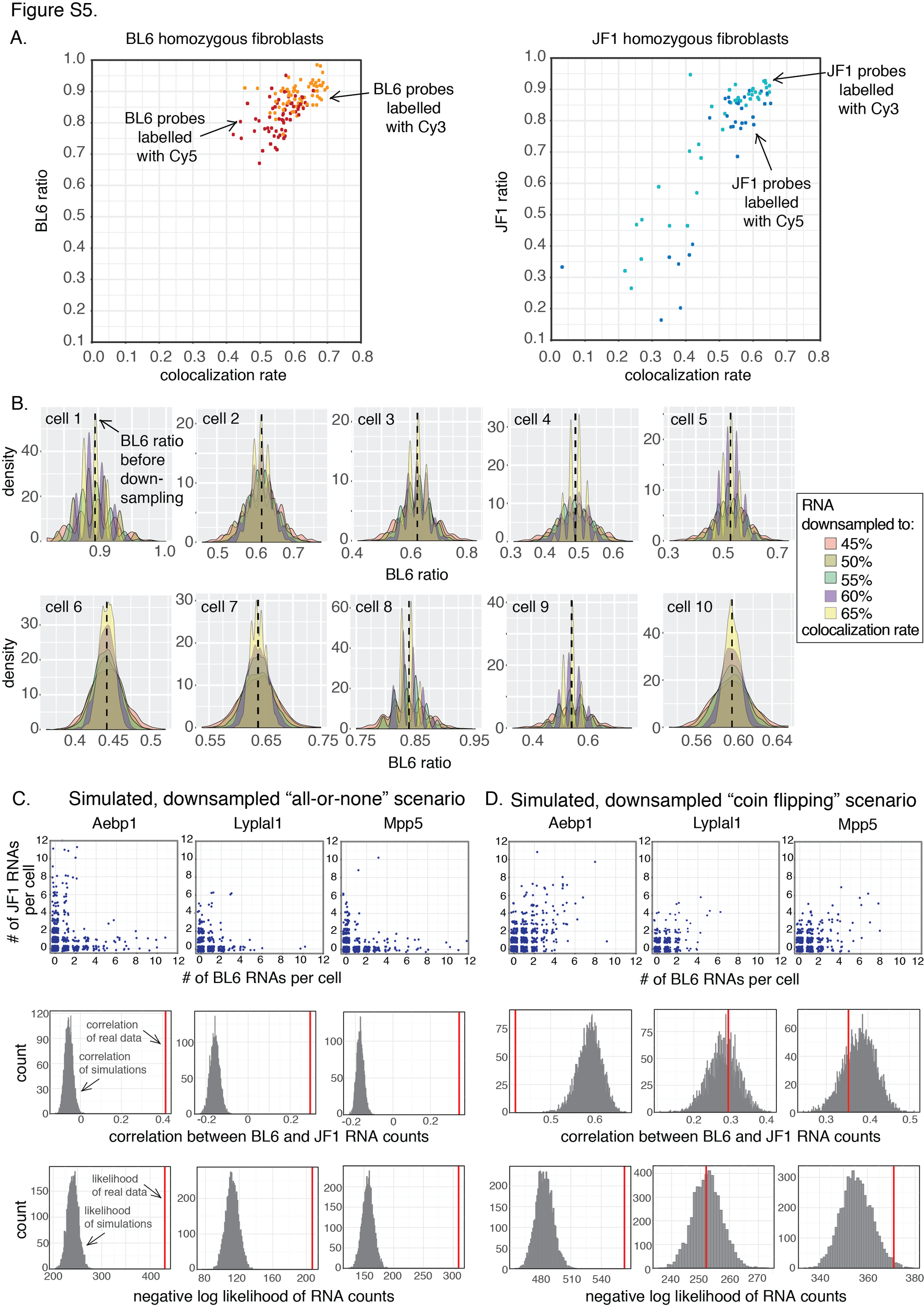

Supplement: S5 Fig — A. The relationship between colocalization rate and correct assignment rate for RNAs in homozygous fibroblast cells. Each spot represents a measurement from a single cell and different dye combinations are displayed in different colors. B. The effect of colocalization rate on measurement accuracy. Each panel represents a single heterozygous fibroblast cell with >70% colocalization rate. The ratio of BL6 assignments measured in each cell is shown by the dashed line. The density plots show the allelic ratio after randomly downsampling the original RNAs to the indicated colocalization rate. C, D. Testing the effect of colocalization rate on “all-or-nothing” (C) and “coin flipping” (D) simulations. First, simulated cells were generated where the total number of RNAs was assigned an identity according to the model of interest. Next, the RNA in each cell was randomly downsampled to the number of RNAs that had been assigned a unique BL6 or JF1 identity in the original measurement. Each simulation was performed 5000 times. The simulated single-cell RNA counts from a randomly selected simulation are shown (top), as well as the correlations (middle) and log likelihoods (bottom) calculated for the entire population. (TIF) [file pgen.1007874.s005.tif]

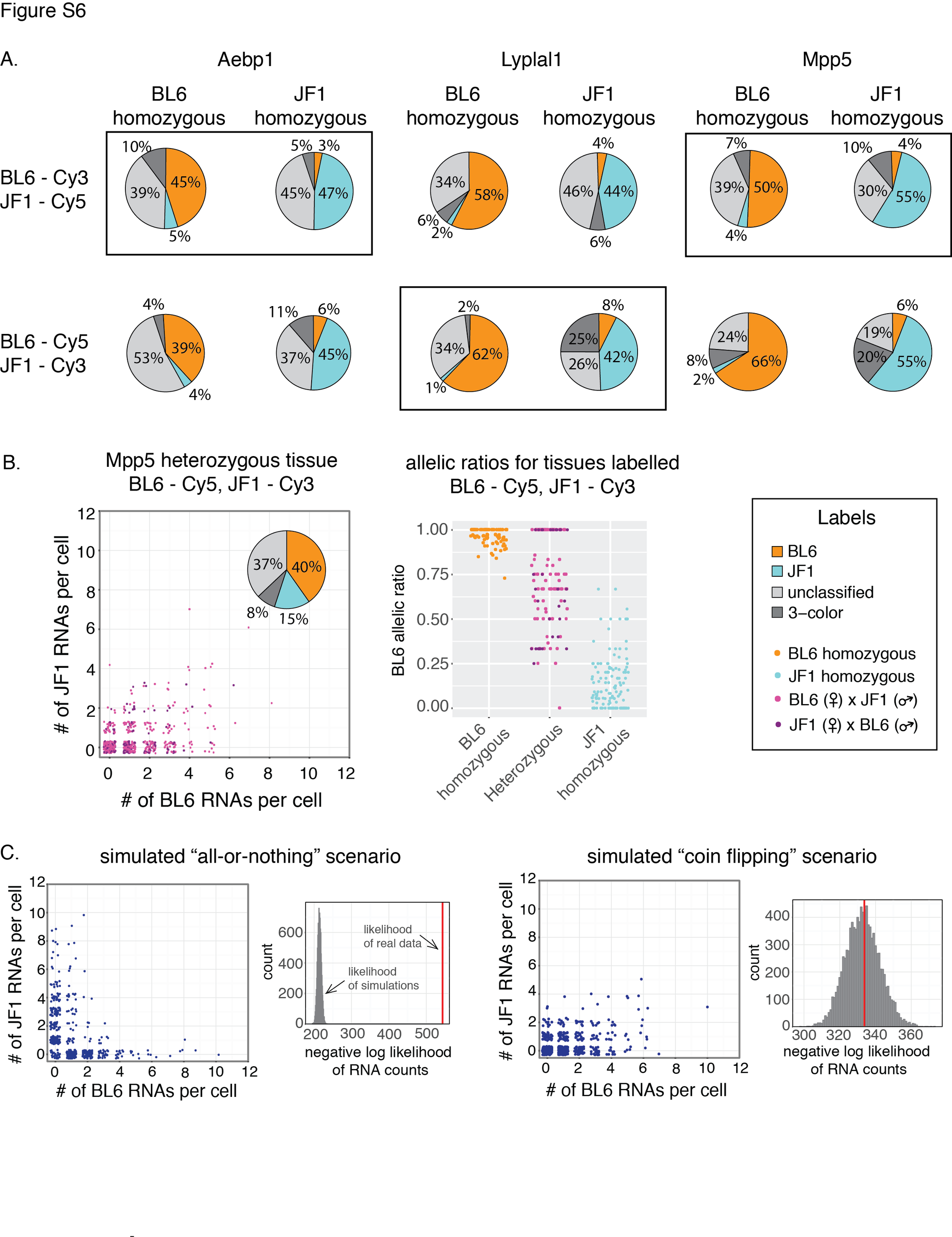

Supplement: S6 Fig — A. Measurements of allele-specific expression in bulk data for Aebp1, Lyplal1 and Mpp5 homozygous tissues. For each gene, allele-specific probes were either labelled with Cy3 for BL6 and Cy5 for JF1 (top row) or Cy5 for BL6 and Cy3 for JF1 (bottom row). Boxes indicate the dye combination used for subsequent experiments, and the pie charts shown in those boxes are the same as displayed in Fig 2B, 2D and 2F. For all plots we combined data from different replicates with more than 40% colocalization rate. B. Single-cell allele-specific expression for Mpp5 in heterozygous tissue and allelic ratios detected in single-cell and bulk tissue data for heterozygous and homozygous tissues. The cells on the heterozygous scatter plot only contained integer numbers of RNA, but we included jitter to better display the density of cells with a given allelic distribution. The two colors on the heterozygous plots from the two reciprocal crosses (i.e. BL6 x JF1 and JF1 x BL6). C. Results of simulations for heterozygous Mpp5 data: for both “all-or-none” and “coin-flip” model we show simulated single-cell RNA counts (left) and negative log likelihood distribution of simulated and observed data (right). (TIF) [file pgen.1007874.s006.tif]

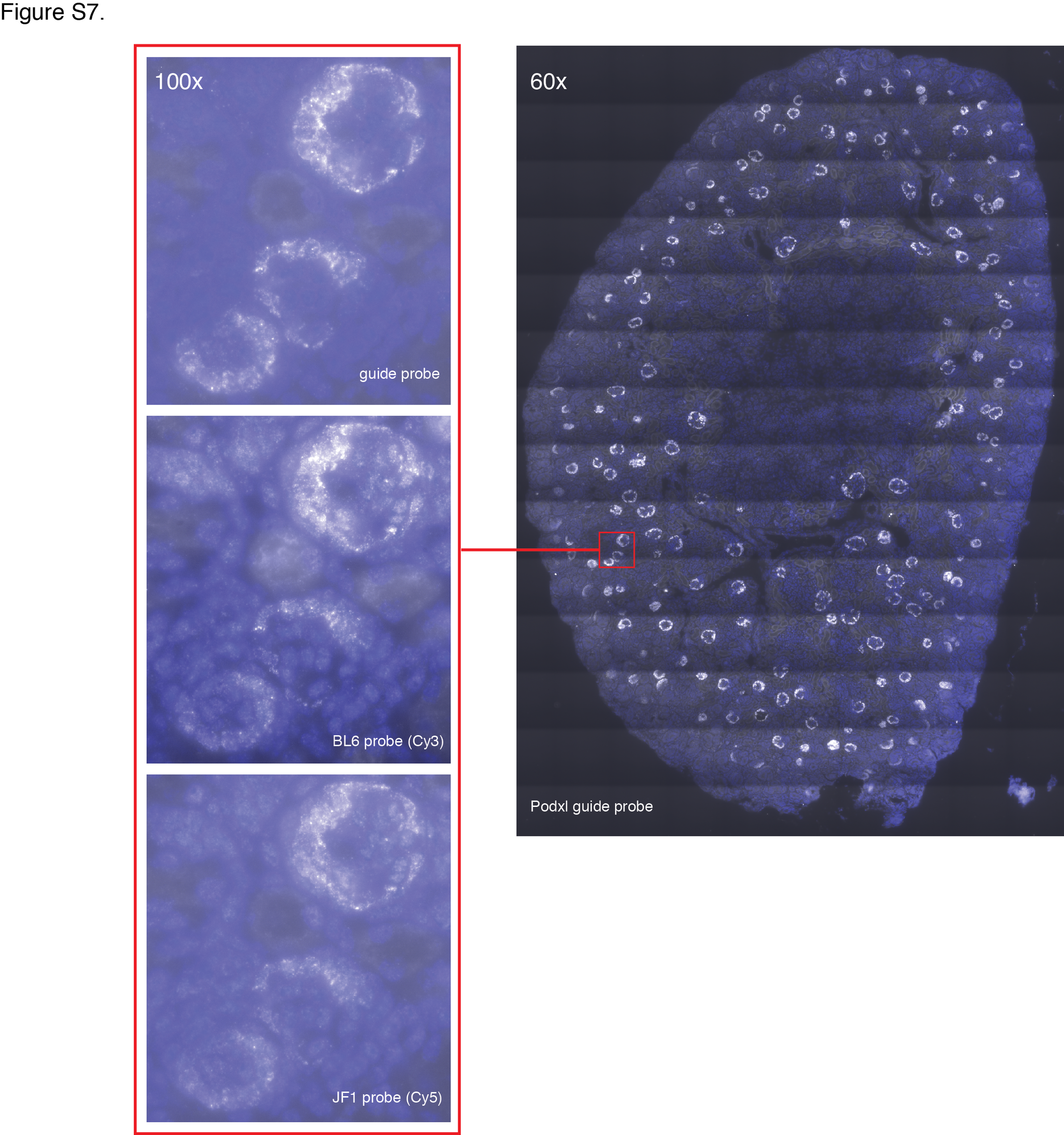

Supplement: S7 Fig — A tissue scan of the Podxl guide probe (labelled with Cal fluor 610) shows staining primarily in glomeruli, due to expression of the gene in podocytes (right). At 100x resolution these areas of high expression can clearly be distinguished from background when detecting fluorescence from the guide probe (left, top), as well as for the BL6 (left, middle) and JF1-specific probes (left, bottom). However, the high expression levels of the gene precludes the precise thresholding and detection of individual mRNA spots. (TIF) [file pgen.1007874.s007.tif]

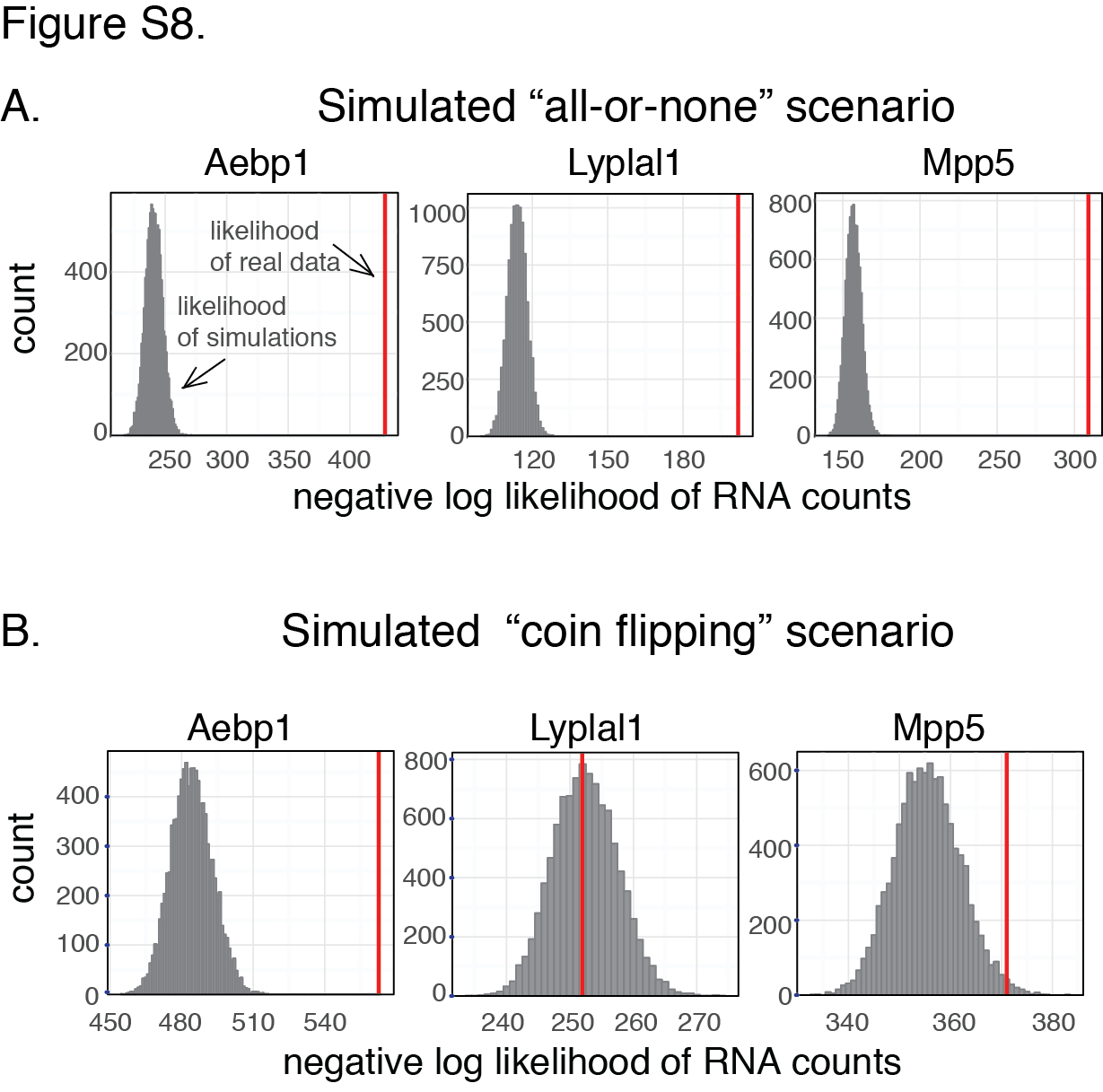

Supplement: S8 Fig — Log likelihood distributions of “all-or-none” (A) and “coin flipping” simulations (B). We show the negative log likelihoods calculated for 10,000 simulations for each gene, as well as the log likelihood of the real data (red line) using the same model as for the simulations. (TIF) [file pgen.1007874.s008.tif]

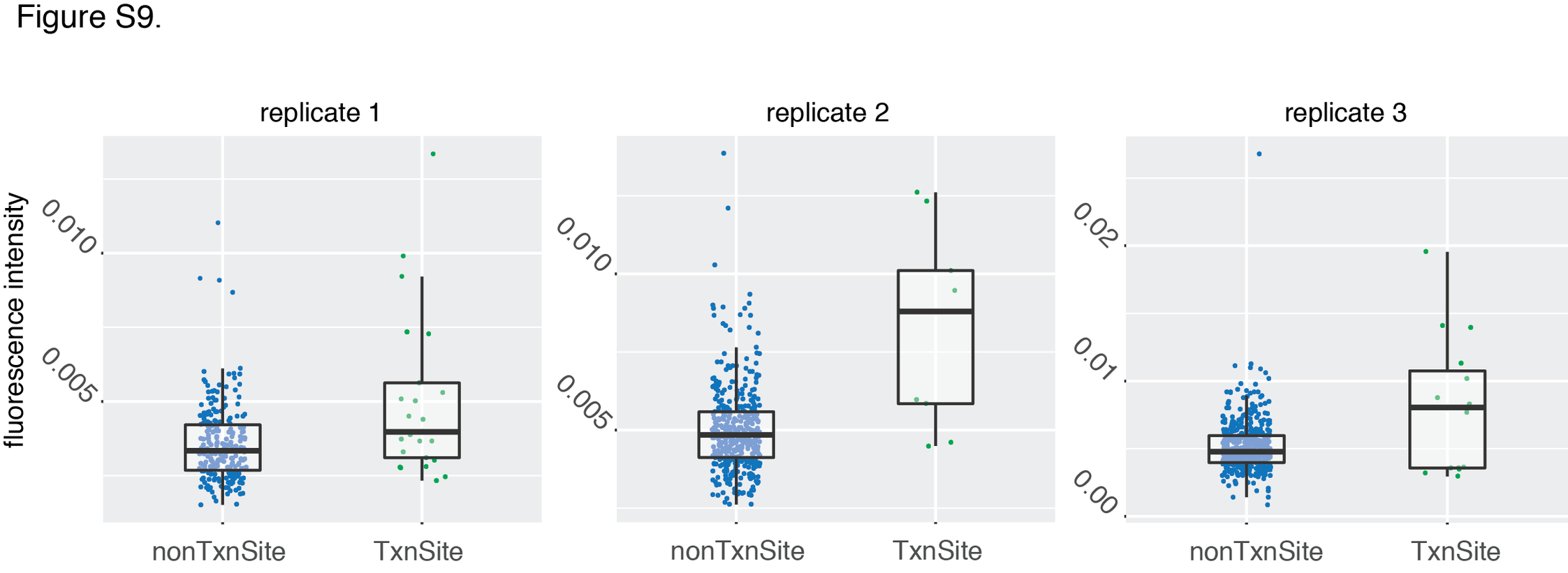

Supplement: S9 Fig — Each spot represents the intensity of a single mRNA guide spot (labelled with Cal fluor 610). Transcription sites were identified by overlap with intron probes labelled with Atto488 (replicate 1) or Alexa700 (replicate 2 and 3). (TIF) [file pgen.1007874.s009.tif]

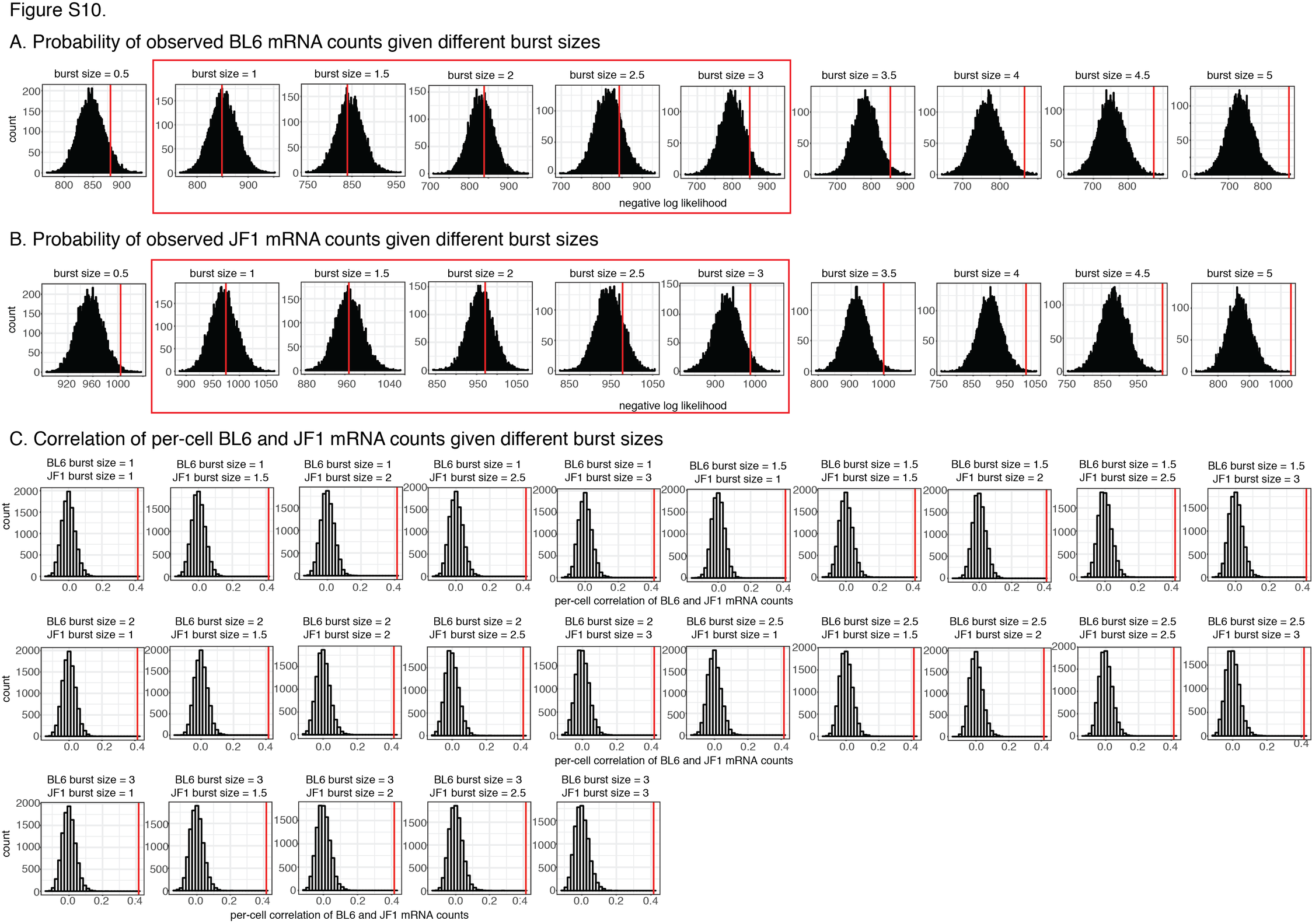

Supplement: S10 Fig — A, B. Probability of observed BL6 (A) and JF1 (B) mRNA counts given different burst sizes. Burst sizes boxed in red showed a good fit for the two alleles individually and were used for correlation analysis. C. Correlation of per-cell BL6 and JF1 mRNA counts given the burst sizes that showed a good fit (boxed in red in A and B). Burst sizes of the alleles are indicated, and for each correlation analysis the simulations for BL6 and JF1 mRNA counts were paired up randomly. In all figures bar plots represent simulations, red line represents real data. (TIF) [file pgen.1007874.s010.tif]

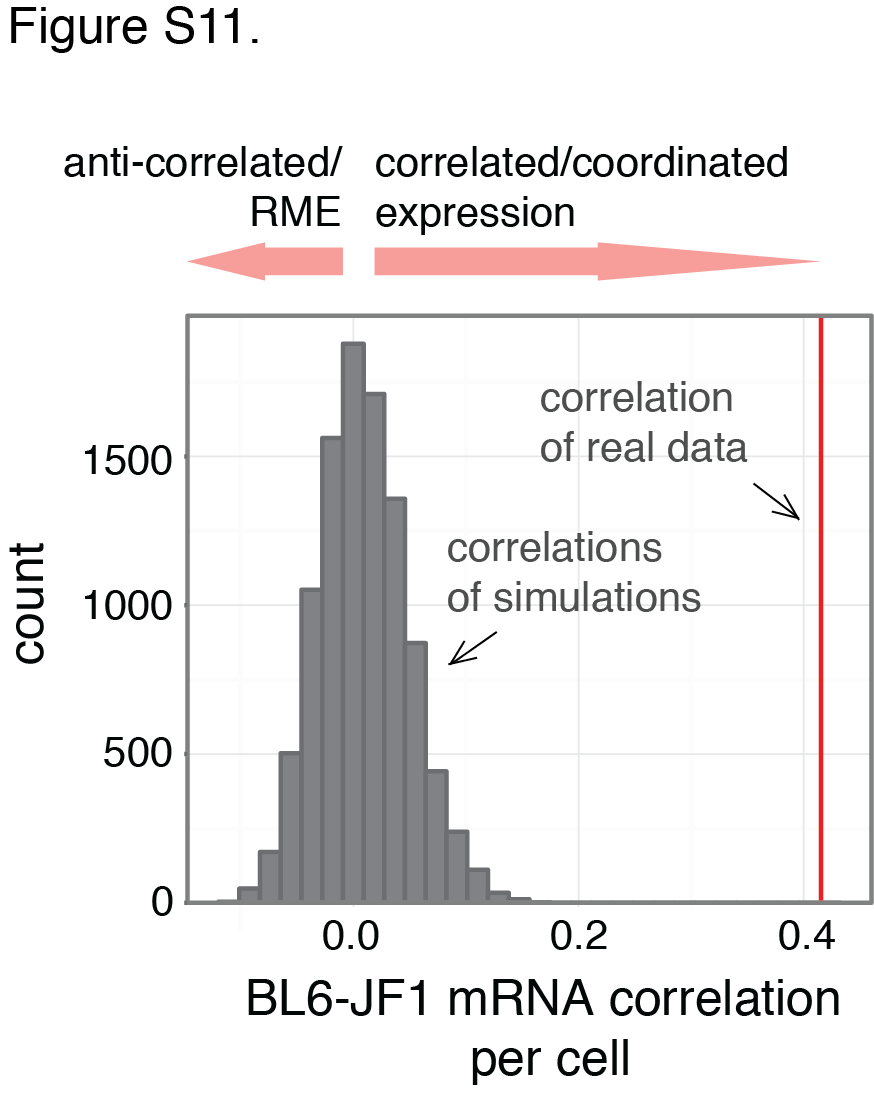

Supplement: S11 Fig — Correlation values for simulated counts in the case independent bursting from the two Aebp1 alleles is shown as grey bar plot, correlation for real data is indicated as red line. (TIF) [file pgen.1007874.s011.tif]
